# Association between media use and poor sleep quality among senior high school students: a cross-sectional study

**DOI:** 10.12688/f1000research.54818.1

**Published:** 2021-11-05

**Authors:** Wisit Chaveepojnkamjorn, Jutiporn Srikaew, Pratana Satitvipawee, Supachai Pitikultang, Soontaree Khampeng

**Affiliations:** 1 Department of Epidemiology, Faculty of Public Health, Mahidol University, Ratchathewi, Bangkok, 10400, Thailand; 2 Ratchaburi Provincial Public Health Office, Ministry of Public Health, Ratchaburi, 70000, Thailand; 3 Department of Biostatistics, Faculty of Public Health, Mahidol University, Ratchathewi, Bangkok, 10400, Thailand; 4 Department of Family Health, Faculty of Public Health, Mahidol University, Ratchathewi, Bangkok, 10400, Thailand; 5 Boromarajonani College of Nursing Phraphutthabat, Ministry of Public Health, Saraburi, 18120, Thailand

**Keywords:** media use, poor sleep quality, senior high school students

## Abstract

**Background *:*** Poor sleep quality (PSQ) is an increasing health problem among adolescents. Mobile phones and portable media devices have become a part of children’s lives and may affect their sleep duration and quality. This study aimed to explore the prevalence of PSQ and identify the association between media use and PSQ among adolescents studying in high school grades 10-12.

**Methods:** This cross-sectional study was conducted in central Thailand. A multi-stage sampling technique was used to enroll 777 adolescents from eight schools from August to October 2016. The research instruments comprised factors of demographics and consumption behaviors and the Pittsburgh Sleep Quality Index questionnaire. Multivariable logistic regression was used to calculate adjusted odds ratios (OR _adj_) and 95% confidence intervals (CI).

**Results:** Prevalence of PSQ was 56.24%. The study subjects were mostly 16-17 years old (67.82%) and female (70.39%). Multivariable logistic regression, after controlling for possible confounders, revealed an increased odds of PSQ among those who used a social media device (OR=1.34, 95%CI=0.97-1.87), and showed a higher proportion of social media use in the PSQ group.

**Conclusion:** A surveillance system to detect media use and PSQ should be conducted accompanied by knowledge sharing on media use among parents, teachers and adolescents. To determine causal relationships, further longitudinal studies will be required to test the association between media users and PSQ. This study may also provide some implications for health promotion on sleep quality of senior high school students.

## Abbreviations

CMB: the China Medical Board

GPA: grade point average

GSQ: Good Sleep Quality

OR _adj_: adjusted odds ratio

OR _c_: crude odds ratio

PSQ: Poor Sleep Quality

PSQI: the Pittsburgh Sleep Quality Index

## Introduction

Sleep is an essential part of life and plays important roles in physical and mental health. ^1^^,^^2^ Adolescents experience significant changes to the body and mind associated with sex hormones. ^3^ Insufficient sleep has been one of the most important public health problems among adolescents. Concerning the aspect of sleep, a few studies have found that poor sleep quality (PSQ) was associated with the amount of daytime sleep, exhaustion, weight gain, obesity, impaired memory and motor vehicle accidents. ^4^^–^^6^ PSQ is currently a widespread issue in most societies. The prevalence of PSQ among adolescents was reported to range from 32 to 62% ^7^^–^^11^ reflecting a wider range of PSQ prevalence. In Thailand, the prevalence of PSQ among adolescents was reportedly 32 to 48%. ^7^^,^^8^ Insufficient sleep not only impacts at a personal level, but also can cause major impact on a larger scale through a high *burden* of non-communicable diseases, ^12^ many events such as motor vehicle crashes, ^13^ workplace accidents, increased mortality and reduced quality of life. ^14^ Media use such as watching TV and using electronic devices are activities that cause PSQ among children and adolescents. Especially among school age group, having a TV in the bedroom can disturb sleep resulting in decreased sleep duration and insufficient sleep. In addition, media use may increase the activity of physiological arousal, inadequate sleep hygiene practice and difficulty falling asleep. ^11^ Some studies have shown the association of media use related to PSQ. ^15^^–^^17^ Hence, the present study aimed to seek the prevalence of PSQ and determine its association with media use among senior high school students in Ratchaburi Province, Thailand. Provinces of Western Thailand which are composed of Kanchanaburi, Phetchaburi, Prachuap Khiri Khan, Tak and Ratchaburi, geographical region and academic area are similar so the authors selected Ratchaburi Province as the area of study because of its characteristics as a proxy of western provinces of Thailand. Ratchaburi is located on the bank of the Mae Klong river and one of the western provinces of Thailand with an area of about 5,196 square kilometer. It lies 80 km west of Bangkok, and borders Myanmar to the west with the Tenasserim Hills as a natural border containing a population of 871,714 and density of 170 km ^2^ in 2017. ^18^^,^^19^

## Methods

### Study design and participants

A cross-sectional study was carried out between August and October 2016 to explore PSQ and identify the association between media use and PSQ occurrence among senior high school students in Ratchaburi Province, Thailand.

### Sample size and sampling technique

The sample size was calculated using a formula to estimate the population proportion with specified absolute precision ^20^ according to the following assumption: 32% of PSQ among adolescents (P), ^7^ with 95% confidence interval and 5% specified absolute precision (d). As a multistage sampling technique was employed to identify study subjects, a design effect of 2 was used. The calculated sample size totaled 709. Also, approximately 10% was added to adjust for nonresponses. Thus, the final sample size was at least 777.

A multi-stage stratified sampling technique was used to identify study subjects from senior high schools in Ratchaburi Province. Schools were stratified by student numbers, namely, extra large (>2,500), large (1,500-2,499) and medium (500-1,499). We randomly selected at least one school from the list of three school categories: urban and rural public schools and private schools. The selection of schools was based on a list of schools obtained from the Provincial Education Office and willingness of school administrators to participate in the study. For each of the schools, the student sample size was calculated proportional to the size of the schools.

### Ethical approval

The study was conducted in accordance with the ethical principles in the Declaration of Helsinki, and the protocol was reviewed and approved by the Human Research Ethical Review Committee of the Faculty of Public Health, Mahidol University (COA. No. MUPH 2016-097). The purpose of this study was explained to school principals and teachers of the target schools. Permission was obtained from these schools and students; written informed consent was obtained from the student’s parents or legal guardians after informing them of the study details (the objectives of study, methods and protection of human rights). Parents or legal guardians were told that participating in the survey was voluntary and that the survey would remain anonymous. Confidentiality was maintained throughout the study using anonymous technique (schools and respondents were identified by code numbers to ensure confidentiality and the results were analyzed as a whole group).

### Procedures

Study population was senior high school students grades 10-12 during the educational year 2016 in Ratchaburi province.

### Inclusion criteria

a) Students who studied in grades 10-12.
b) Students who studied in high schools that were under the control of the secondary education service area office 8, Ratchaburi province
c) Students who were willing to participate in the study and provided the written informed consent.
d) Students who provided the written informed consent signed by their parents or legal guardians.

### Exclusion criteria

a) Students who were absent from school on a period of data collection.
b) Students who were chronically ill during the time of study.

Researchers contacted the educational administrators and the teachers for data collection. The paper-based questionnaire was provided for the participants to fill data at the free time from studying at their school. Researcher and research assistants explained the details of questionnaire and answered the questions from participants. This process was approximately 40 minutes. Information was collected using a self-administered, anonymous questionnaire comprising three parts, namely, demographics, consumption behaviors relating to sleep quality, sleep quality assessment and media-used evaluation. A copy of the questionnaire can be found in the *Extended data*. ^35^ Sleep quality was evaluated using the Pittsburgh Sleep Quality Index (PSQI) translated to Thai with a cutoff point of scores > 5 was classified as poor sleepers and ≤5 was classified as good sleepers. ^20^^–^^23^ Reliability was tested revealing a Cronbach’s alpha of 0.86.

### Data analysis

The data were encoded and processed for statistical analysis by using SPSS for Windows, Version 18). Categorical variables were given as frequency and percentage, crude odds ratio (OR _c_), 95 % CI of OR and p-value. Moreover, numerical variables were expressed as mean, median, minimum and maximum, standard deviation and quartile deviation. Univariate analysis was performed using univariable logistic regression to differentiate proportional exposures between poor and good sleepers for categorical variables. Adjusted odds ratio (OR _adj_) and 95% CI of OR were calculated from multivariable logistic regression to examine associations between media use and PSQ occurrence, adjusted for potential confounders. All statistics were performed using two-sided tests, and the criteria of p <0.05 was judged to be statistically significant.

## Results

### Demographic data of participants

In total, 777 students were selected for the present study. The majority were female (70.39%), aged 16 to 17 years (67.82%), studying in Grade 12 (35.39%), GPA 3.01 to 3.50 (40.14%), monthly family income ≤10,000 THB (44.67%), no smoking (98.33%) and no alcohol consumption (85.33%), as shown in Table 1.

**Table 1.** Demographic characteristics of senior high school students.

Variables	No. (%)
Sex (n = 777)	
Female	547 (70.39)
Male	230 (29.61)
Age (year) (n = 777)	
<16	123 (15.83)
16-17	527 (67.82)
>17	127 (16.35)
Mean (SD)	16.51 (0.96)
Min-Max	14-19
Education level (Grade) (n = 777)	
10	247 (31.79)
11	255 (32.82)
12	275 (35.39)
Parental marital status (n = 777)	
Married	517 (66.54)
Widowed, divorced, separated	260 (33.46)
Monthly family income (THB) (n = 647)	
≤10,000	289 (44.67)
10,001-30,000	280 (43.28)
30,001-50,000	47 (7.26)
50,001-70,000	9 (1.39)
>70,000	22 (3.40)
Median	10,000
Min-Max	1,800-300,000
Grade point average (n = 715)	
<2.50	58 ( 8.11)
2.51-3.00	242 (33.85)
3.01-3.50	287 (40.14)
≥3.50	128 (17.90)
Mean (SD)	3.09 (0.42)
Median (QD)	3.10 (0.29)
Min-Max	1.33 – 3.99
Underlying diseases (n = 777)	
No	668 (85.97)
Yes	109 (14.03)
Smoking (n = 774)	
No	764 (98.33)
Yes	10 ( 1.67)
Alcohol consumption (n = 777)	
No	663 (85.33)
Yes	114 (14.67)
Illness history during last month (n = 777)	
No	537 (69.11)
Yes	240 (30.89)

### PSQ and associated factors

The prevalence of PSQ was 56.24%. Using univariable logistic regression analysis, associated demographic factors of PSQ among adolescents included illness history during the last month, coffee and tea consumption, reading, annoyance, poor ventilation, stress, depression and sleep duration (p < 0.05), as shown in Table 2. In case of media use, we found an association between social media use and PSQ (OR = 1.53, 95%CI = 1.13-2.08), as shown in Table 3. Using multivariable logistic regression analysis, regarding association between social media use and PSQ among adolescents (adjusted for potential confounders), social media users were 1.34 times at risk compared with those of nonusers (OR = 1.34, 95%CI = 0.97-1.87) but without significance, as shown in Table 4. Comparing PSQ and good sleep quality (GSQ) groups, the most commonly activity before bedtime was social media (44.56%, 37.38%) and television watching (20.78%, 30.29%) respectively. Further, we found a higher proportion of social media use in the PSQ group, as shown in Table 5.

**Table 2.** Univariable logistic regression analysis of factors associated with PSQ among senior high school students.

Variables	Poor sleep quality/total	%	ORc	95%CI	p-value
Age group (year) (n = 777)					
<16	59/123	47.97	1		
16-17	303/527	57.49	1.47	0.99-2.18	0.056
>17	75/127	59.06	1.57	0.95-2.58	0.079
Sex (n = 777)					
Female	308/547	56.31	1		
Male	129/230	56.09	0.99	0.72-1.37	0.982
Education level (Grade) (n = 777)					
10	131/247	53.04	1		
11	148/255	58.04	0.84	0.59-1.19	0.323
12	156/275	56.73	1.03	0.73-1.45	0.873
Parental marital status (n = 774)					
Married	284/517	54.93	1		
Widowed, divorced, Separated	151/257	58.75	0.86	0.62-1.17	0.351
Family members (n = 629)					
Father and mother	271/346	78.32	1		
Father or mother only	166/283	58.66	1.16	0.87-1.56	0.319
Relative/Friend					
Monthly family income (THB) (n = 647)					
≤10,000	171/289	59.17	1	0.52-1.05	0.091
10,001-30,000	145/280	51.79	0.74	0.52-2.00	0.914
30,001 **-**50,000	28/47	59.57	1.02	0.43-2.16	0.942
>50,000	18/31	58.06	0.96		
Grade point average (n = 715)					
≥3.50	76/128	59.38	1		
3.01-3.50	146/287	50.87	1.26	0.89-1.78	0.188
2.51-3.00	137/242	19.01	1.24	0.72-2.25	0.403
<2.50	33/58	50.00	1.41	0.93-2.15	0.109
Underlying diseases (n = 777)					
No	370/668	55.39	1		
Yes	67/109	61.47	1.29	0.85-1.95	0.279
Smoking (n = 774)					
No	430/764	56.28	1		
Yes	7/10	70.00	1.81	0.47-7.06	0.527
Alcohol consumption (n = 777)					
No	368/663	55.51	1		
Yes	69/114	60.53	1.23	0.82-1.84	0.318
Illness history during the last month (n = 777)					
No	283/537	52.70	1		
Yes	154/240	64.17	1.61	1.18-2.20	<0.001 *
Coffee consumption (n = 777)					
No	385/702	54.84	1		
Yes	52/75	69.33	1.86	1.12-3.10	0.022 *
Tea consumption (n = 777)					
No	216/412	52.43	1		
Yes	221/365	60.55	1.39	1.05-1.85	0.027 *
Reading (n = 777)					
No	428/751	56.99	1		
Yes	9/26	34.62	0.40	0.18-0.91	0.024 *
Annoyance (n = 777)					
No	352/658	53.49	1		
Yes	85/119	71.43	2.17	1.39-3.41	<0.001 *
Poor ventilation (n = 777)					
No	393/719	54.66	1		
Yes	44/58	75.86	2.61	1.36-5.08	0.002 *
Stress (n = 777)					
No	69/195	35.38	1		
Yes	368/582	63.23	3.14	2.24-4.41	<0.001 *
Depression (n = 777)					
No	276/565	35.38	1		
Yes	161/212	63.23	3.31	2.32-4.72	<0.001 *
Sleep duration (hrs) (n = 777)					
>7	54/254	21.26	1		
6-7	297/435	68.28	19.98	5.21-169.49	<0.001 *
<6	86/88	97.73	159.26	39.82-1354.78	<0.001 *

ORc = crude odds ratio, CI = confidence interval.

* Statistically significant (p < 0.05).

**Table 3.** Univariable logistic regression analysis of media use associated with PSQ among senior high school students.

Variables	Poor sleep quality/total	%	ORc	95%CI	p-value
Video gaming (n = 777)					
No	386/690	55.94	1		
Yes	51/87	58.62	1.12	0.71-1.75	0.635
Phone calling (n = 777)					
No	402/724	55.52	1		
Yes	35/53	66.04	1.56	0.87-2.80	0.139
Music listening (n = 777)					
No	396/707	56.01	1		
Yes	41/70	58.57	1.11	0.67-1.83	0.681
Social media use (n = 777)					
No	274/519	52.79	1		
Yes	163/258	63.18	1.53	1.13-2.08	0.006 *
Television watching (n = 777)					
No	361/624	57.85	1		
Yes	76/153	49.67	0.72	0.50-1.03	0.068

ORc = crude odds ratio, CI = confidence interval.

* Statistically significant (p < 0.05).

**Table 4.** Multivariable logistic regression of social media use associated with PSQ among senior high school students.

Variables	ORc	95%CI	ORadj	95%CI	p-value
Social media use					
No	1		1		
Yes	1.53	1.13-2.08	1.34	0.97-1.87	0.079

ORc = crude odds ratio, ORadj = adjusted odds ratio for illness history during the last month, coffee consumption, tea consumption, reading, annoyance, poor ventilation, stress and depression.

**Table 5.** Percent of media use before bedtime.

Variables	PSQ (%)	GSQ (%)
Social media use	44.56	37.38
Television watching	20.78	30.29
Video Gaming	13.94	14.17
Music listening	11.20	11.41
Phone calling	9.52	6.75

PSQ = poor sleep quality, GSQ = good sleep quality.

## Discussion

Our findings demonstrated that PSQ prevalence rate was about 56% higher than related studies conducted in Thailand. ^7^^,^^8^ Evidence from related studies on PSQ among college students showed PSQ prevalence was approximately from 32 to 62%. ^7^^–^^11^ The difference of PSQ occurrence might have stemmed from various factors, namely, environment, lifestyle, household characteristics, social media and activities, health behaviors etc. Univariable analysis showed that social media use played a critical role in the development of PSQ among adolescents (OR = 1.53, p = 0.006). However multivariable logistic regression analysis did not indicate significant differences (OR = 1.34, 95%CI = 0.97-1.87). Some studies indicated adolescents who used social media before bedtime had lower sleep efficiency. ^15^^–^^17^^,^^24^^–^^27^ Mobile phone use among young students for daily calling, using e-mail, text messaging and social network services were associated with short sleep duration, PSQ, excessive daytime sleepiness and presenting insomnia symptoms. ^15^^,^^28^^,^^29^ Higher frequency and volume of social media use had significantly greater odds of having sleep disturbance among young adults, ^24^^,^^26^ while one study showed a better sleep quality among users. ^30^ The present study showed the prevalence of social media use before bedtime in the PSQ group was approximately 44.56%. One half of social media users spent over 2 hours per day. The average time for social media use was 3.58 hours per day, and this might have affected sleep pattern. A related study showed users who spent 0.5 to 2 hours per day on social media were more likely to have poor sleep than those of spent less than 0.5 hours. ^31^ In addition, the meta-analysis studies reported social media users before bed were more likely to have insufficient sleep and tended to have PSQ. ^27^^,^^32^ Some related studies have suggested blue light emitted from smart phones might disturb sleep. ^33^^,^^34^ Therefore, monitoring social media use among adolescents, and cooperating with parents, caregivers, teachers and the adolescents themselves is recommended to reduce PSQ problems.

### Study limitations

This study encountered a few limitations that need to be addressed. First, cross-sectional surveys reduced the ability of the study to make direct causal inferences. Second, these data apply only to those aged 14-19 years as the study subjects; therefore, they could not represent all adolescents. Moreover, data collection might have excluded subjects absent from schools. Finally, all data were based using a self-report method subject to recall bias.

## Conclusion

PSQ surveillance systems should be established along with knowledge sharing programs regarding associated factors of PSQ among adolescents with their parents and teachers. We recommend that the use of media and the presence of media equipment in bedroom should be limited. This may be beneficial to sleep quality.

## Data availability

### Underlying data

OSF: Association between media use and poor sleep quality among senior high school students: a cross-sectional study. https://doi.org/10.17605/OSF.IO/KV2BJ. ^35^

This project includes the following underlying data.

- SAV Dataset

### Extended data

OSF: Association between media use and poor sleep quality among senior high school students: a cross-sectional study. https://doi.org/10.17605/OSF.IO/KV2BJ. ^35^

This project includes the following extended data.

- Appendix A (The certificate of ethical approval)
- Appendix B (A copy of the questionnaire)

Data are available under the terms of the Creative Commons Zero “No rights reserved” data waiver (CC0 1.0 Public domain dedication).
