# Supplementary material for: Association between media use and poor sleep quality among senior high school students: a cross-sectional study
Source: F1000Res. 2023 Aug 8;10:1116. Originally published 2021 Nov 5. [Version 2] doi: 10.12688/f1000research.54818.2 (PMC10457560; doi:10.12688/f1000research.54818.2)
Supplement: Peer review history

# Peer review: Reviewer response for version 2

**DOI:** 10.5256/f1000research.153227.r195839

**Published:** 2023-08-25

**Authors:** Sumattana Glangkarn

**Affiliations:** 1 Faculty of Public Health, Mahasarakham University, Kantarawichai, Thailand

I have read the version 2 that has been updated from the version 1 comments. It also reveals the limitations of the study due to it being a cross-sectional study and the subjects were not representative of all adolescents. The follow up study should be recommended for further research and expand the target population.

Is the work clearly and accurately presented and does it cite the current literature?

Yes

If applicable, is the statistical analysis and its interpretation appropriate?

Yes

Are all the source data underlying the results available to ensure full reproducibility?

Yes

Is the study design appropriate and is the work technically sound?

Yes

Are the conclusions drawn adequately supported by the results?

Yes

Are sufficient details of methods and analysis provided to allow replication by others?

Yes

Reviewer Expertise:

Community Public Health

I confirm that I have read this submission and believe that I have an appropriate level of expertise to confirm that it is of an acceptable scientific standard.

# Peer review: Reviewer response for version 2

**DOI:** 10.5256/f1000research.153227.r194727

**Published:** 2023-08-15

**Authors:** Pongdech Sarakarn

**Affiliations:** 1 Epidemiology and Biostatistics Department, Faculty of Public Health, Khon Kaen University, Khon Kaen, Thailand

I have already read the updated version, and the revisions are appropiate.

Is the work clearly and accurately presented and does it cite the current literature?

Yes

If applicable, is the statistical analysis and its interpretation appropriate?

Partly

Are all the source data underlying the results available to ensure full reproducibility?

Yes

Is the study design appropriate and is the work technically sound?

Yes

Are the conclusions drawn adequately supported by the results?

Partly

Are sufficient details of methods and analysis provided to allow replication by others?

Partly

Reviewer Expertise:

Statistical method for health modeling

I confirm that I have read this submission and believe that I have an appropriate level of expertise to confirm that it is of an acceptable scientific standard.

# Peer review: Reviewer response for version 1

**DOI:** 10.5256/f1000research.58338.r166408

**Published:** 2023-04-18

**Authors:** Kavita Batra, Ravi Batra, Sidath Kapukotuwa

**Affiliations:** 1 Kirk Kerkorian School of Medicine at UNLV, University of Nevada, Las Vegas, USA; 2 Social and Behavioral Health, University of Nevada Las Vegas, Las Vegas, NV, USA

1. Title: Please consider adding the place of the study in the title.
2. Introduction: The introduction section is not backed up by sufficient literature review. Relevant references can be added to strengthen the argument of sleep problems among countries with similar standing. Comparison with other developed countries is highly advised.
3. Data are too old. Several things have been changed now especially with the COVID-19 pandemic.
4. Since authors translated the tool in another language, it becomes critical to assess the psychometric validity of the tool. Please describe.
5. What estimates did the authors use to perform stratified sampling? After the stratification, how did the authors ensure random selection?
6. Please do the bivariate group comparisons via Chi-square first.
7. Expand on the potential value of the study.

Is the work clearly and accurately presented and does it cite the current literature?

Partly

If applicable, is the statistical analysis and its interpretation appropriate?

Partly

Are all the source data underlying the results available to ensure full reproducibility?

Partly

Is the study design appropriate and is the work technically sound?

Partly

Are the conclusions drawn adequately supported by the results?

Partly

Are sufficient details of methods and analysis provided to allow replication by others?

Partly

Reviewer Expertise:

Global health,  Health behaviors Research, Maternal and Child Health, Statistics, Epidemiology, COVID-19

We confirm that we have read this submission and believe that we have an appropriate level of expertise to confirm that it is of an acceptable scientific standard, however we have significant reservations, as outlined above.

## References

[1] : Social Media, Stress and Sleep Deprivation: A Triple “S” Among Adolescents.2021;6(2) :159-166 Reference source

# Response

**Published:** 2023-04-21

**Authors:** Wisit Chaveepojnkamjorn

**Affiliations:** Department of Epidemiology, Faculty of Public Health, Mahidol University, Thailand

Respose to this report:

1. We believe that the title is appropriate and concise. The place of study is showed in the methods.
2. The introduction indicated burden of poor sleep quality and media use.
3. The authors studied this study during August-October 2016 and submitted this article on 1 ^st^ July 2021 for publication in the F1000Research.
4. We believed in the tool PSQI version Thai, it is a good tool which we had reference 23.
5. We used the multi stage stratified sampling technique (we added the design effect of 2 and added 10% for non-response).
6. We used the univariable logistic regression for bivariate analysis.

# Peer review: Reviewer response for version 1

**DOI:** 10.5256/f1000research.58338.r99456

**Published:** 2021-11-25

**Authors:** Pongdech Sarakarn

**Affiliations:** 1 Epidemiology and Biostatistics Department, Faculty of Public Health, Khon Kaen University, Khon Kaen, Thailand

This manuscript addresses a very important issue for solving the problem of the adolescent group. However, some components should be considered and added up for fulfilling the content to implication or implementation. I have the following comments:

1. The gap of knowledge for the association between media use and poor sleep quality (PSQ) among senior high school students should be identified clearly. For example, the magnitude of related factors for such an association, in previous studies, have not been justified based on some limitations (conditions). This study can add something more regarding detecting the precision or properly-related factors compared to previous studies
2. Media use is a key factor – I would like to know how it is associated with the PSQ in this study, but the authors didn’t mention the factor in the manuscript. Therefore, the media use should be identified clearly in the methods part.
3. Potential confounders which are included in the multi-variable analysis step should also be identified in the methods section. According to such confounders, some studies (Fatima *et al.*, 2016 ^1^, Galland *et al.*, 2017 ^2^) pointed out that the PSQ had a higher prevalence in girls than in boys. From the results, what do you think about the gender influence in your study? Why didn’t you add the factor as confounder in the model? Or, can you expand more on the subject of gender variables?
4. For explicitly identifying the magnitude of estimation, the 95% CI of the PSQ prevalence should be added.
5. In Table 5, I am confused regarding PSQ and good sleep quality (GSQ) for each type of media use. Given that they are opposites, a table comparing them may be difficult to interpret and lead to misunderstanding. Also, the total of each row doesn’t add up to 100%, could you please explain how you want to want to present this table, and how you calculate the percentage of both (PSQ and GSQ) in each type of the media use? I suggest that the column of GSQ should be removed from the table, because the remainder of PSQ can be interpreted as GSQ. However, each row cannot be calculated total as 100%, so please describe more for this table.
6. According to the results in Table 3, 4 and 5, data management should be mentioned and described in the part of data analysis, regarding how social media is considered and included into the final model.
7. Issues with the statistical method issues should be mentioned, in particular the finding that is not statistically significant. For example, is the standard logistic regression appropriate for this data or not? As students from the same room may be correlated, while some students from the different room may be independent. Therefore, the statistical methods for correlating the outcome, like the generalised estimating equation (GEE) and logistic regression, should maybe be analysed based on identifying the cluster as with the room
8. Finally, even if the data of this study was collected before the transmission of the COVID-19, the issue of media use can change, and affect the topic. I think the author should mention this point in the discussion or limitation part.

Is the work clearly and accurately presented and does it cite the current literature?

Yes

If applicable, is the statistical analysis and its interpretation appropriate?

Partly

Are all the source data underlying the results available to ensure full reproducibility?

Yes

Is the study design appropriate and is the work technically sound?

Yes

Are the conclusions drawn adequately supported by the results?

Partly

Are sufficient details of methods and analysis provided to allow replication by others?

Partly

Reviewer Expertise:

Statistical method for health modeling

I confirm that I have read this submission and believe that I have an appropriate level of expertise to confirm that it is of an acceptable scientific standard, however I have significant reservations, as outlined above.

## References

[1] : Exploring Gender Difference in Sleep Quality of Young Adults: Findings from a Large Population Study. *Clin Med Res* .2016;14(3-4) : 10.3121/cmr.2016.1338 138-144 10.3121/cmr.2016.1338 28188139PMC5302457

[2] : Gender differences in sleep hygiene practices and sleep quality in New Zealand adolescents aged 15 to 17 years. *Sleep Health* .3(2) : 10.1016/j.sleh.2017.02.001 77-83 10.1016/j.sleh.2017.02.001 28346161

# Response

**Published:** 2021-11-29

**Authors:** Wisit Chaveepojnkamjorn

**Affiliations:** Department of Epidemiology, Faculty of Public Health, Mahidol University, Thailand

User Comment: I am an author of this article.

 This manuscript addresses a very important issue for solving the problem of the adolescent group. However, some components should be considered and added up for fulfilling the content to implication or implementation. I have the following comments:

1. To The gap of knowledge for the association between media use and poor sleep quality (PSQ) among senior high school students should be identified clearly. For example, the magnitude of related factors for such an association, in previous studies, have not been justified based on some limitations (conditions). This study can add something more regarding detecting the precision or properly-related factors compared to previous studies

**Response**

 Thank you very much for your comments. The objectives of study aimed to seek the prevalence of PSQ and determine its association with media use among senior high school students. We justified the definition of PSQ by using the Pittsburgh Sleep Quality Index (PSQI) for poor and good sleepers.

  

1. Media use is a key factor – I would like to know how it is associated with the PSQ in this study, but the authors didn’t mention the factor in the manuscript. Therefore, the media use should be identified clearly in the methods part.

**Response**

 The authors made considerations about the media use, and made the questionnaire for collecting the media use such as watching TV and using electronic devices before bedtime.

  

1. Potential confounders which are included in the multi-variable analysis step should also be identified in the methods section. According to such confounders, some studies (Fatima *et al.*, 2016 ^1^ , Galland *et al.*, 2017 ^2^ ) pointed out that the PSQ had a higher prevalence in girls than in boys. From the results, what do you think about the gender influence in your study? Why didn’t you add the factor as confounder in the model? Or, can you expand more on the subject of gender variables?

**Response**

 Demographic factors associated with PSQ (such as illness history during the last month and etc) were controlled by the multivariable logistic regression. It showed at the end of Table 4. The issue of gender, Table 2 sex variable didn’t show the association with PSQ and we didn’t add this one in the model.

  

1. For explicitly identifying the magnitude of estimation, the 95% CI of the PSQ prevalence should be added.

**Response**

 The PSQ was showed in the result section (PSQ and associated factors) and we are ready added the 95% CI of the PSQ (52.75-59.74).

  

1. In Table 5, I am confused regarding PSQ and good sleep quality (GSQ) for each type of media use. Given that they are opposites, a table comparing them may be difficult to interpret and lead to misunderstanding. Also, the total of each row doesn’t add up to 100%, could you please explain how you want to want to present this table, and how you calculate the percentage of both (PSQ and GSQ) in each type of the media use? I suggest that the column of GSQ should be removed from the table, because the remainder of PSQ can be interpreted as GSQ. However, each row cannot be calculated total as 100%, so please describe more for this table.

**Response**

 Table 5 showed the proportion of media use among the PSQ and the GSQ. We would like to seek the difference of the media use in both groups.

  

1. According to the results in Table 3, 4 and 5, data management should be mentioned and described in the part of data analysis, regarding how social media is considered and included into the final model.

**Response**

 We are ready added your recommendations.

 The data were verified, encoded and processed…

 We added at the end of potential confounders (enter method)

  

1. Issues with the statistical method issues should be mentioned, in particular the finding that is not statistically significant. For example, is the standard logistic regression appropriate for this data or not? As students from the same room may be correlated, while some students from the different room may be independent. Therefore, the statistical methods for correlating the outcome, like the generalised estimating equation (GEE) and logistic regression, should maybe be analysed based on identifying the cluster as with the room

**Response**

 We concerned and realized of the statistical use for analysis. Statistical methods such as logistic regression is a tool to prove the association between the exposure and the outcome. However, every statistics had its limitation and criteria for appropriate use.

  

1. Finally, even if the data of this study was collected before the transmission of the COVID-19, the issue of media use can change, and affect the topic. I think the author should mention this point in the discussion or limitation part.

**Response**

 We think the pandemic of COVID-19 changed the learning of everyone . The trend of social media use was rapidly increasing every generation groups . The further studies should be concerned after the COVID-19 pandemic.
